# Salicylic Acid as a Disinfectant

**Published:** 1875-08

**Authors:** 


Salicylic Acid as a Disinfectant.—This agent has been recently introduced into the surgical department of Bellevue Hospital, and serves a much better purpose than carbolic acid. The great advantage it possesses is, that it is destitute of odor, while it thoroughly deodorizes all discharges that it comes in contact with. It is used in solution directly to the granulating surface, by means of a syringe or irrigator. The solution is made by combining and dissolving the following: Salicylic acid one part; phosphate of soda three parts; water one hundred parts.—*New York Medical Journal.*

